# Molecular imaging reporting and data systems (MI-RADS): a generalizable framework for targeted radiotracers with theranostic implications

**DOI:** 10.1007/s12149-018-1291-7

**Published:** 2018-08-14

**Authors:** Rudolf A. Werner, Ralph A. Bundschuh, Lena Bundschuh, Mehrbod S. Javadi, Takahiro Higuchi, Alexander Weich, Sara Sheikhbahaei, Kenneth J. Pienta, Andreas K. Buck, Martin G. Pomper, Michael A. Gorin, Constantin Lapa, Steven P. Rowe

**Affiliations:** 1 The Russell H. Morgan Department of Radiology and Radiological Science, Johns Hopkins University School of Medicine, 601 N. Caroline St., Baltimore, MD 21287 USA; 2 Department of Nuclear Medicine, Comprehensive Heart Failure Center, University Hospital Würzburg, Würzburg, Germany; 3 European Neuroendocrine Tumor Society (ENETS), Center of Excellence (CoE), NET Zentrum, University Hospital Würzburg, Würzburg, Germany; 4 Department of Nuclear Medicine, University Medical Center Bonn, Bonn, Germany; 5 Department of Bio Medical Imaging, National Cardiovascular and Cerebral Research Center, Suita, Japan; 6 Department of Internal Medicine II, Gastroenterology, University Hospital Würzburg, Würzburg, Germany; 7 Department of Urology, The James Buchanan Brady Urological Institute, Johns Hopkins University School of Medicine, Baltimore, MD USA

**Keywords:** Prostate cancer, Neuroendocrine tumor, Prostate-specific membrane antigen (PSMA), Somatostatin receptor (SSTR), Positron emission tomography, Theranostics, Standardization, RADS, Reporting and data systems

## Abstract

Both prostate-specific membrane antigen (PSMA)- and somatostatin receptor (SSTR)-targeted positron emission tomography (PET)-based imaging agents for prostate carcinoma and neuroendocrine tumors, respectively, are seeing rapidly expanding use. In addition to diagnostic applications, both classes of radiotracers can be used to triage patients for theranostic endoradiotherapy. While interpreting PSMA- or SSTR-targeted PET/computed tomography (CT) scans, the reader has to be aware of certain pitfalls. Adding to the complexity of the interpretation of those imaging agents, both normal biodistribution, and also false-positive and -negative findings differ between PSMA- and SSTR-targeted PET radiotracers. Herein summarized under the umbrella term molecular imaging reporting and data systems (MI-RADS), two novel RADS classifications for PSMA- and SSTR-targeted PET imaging are described (PSMA- and SSTR-RADS). Notably, PSMA- and SSTR-RADS are structured in a reciprocal fashion, i.e., if the reader is familiar with one system, the other system can readily be applied, as well. In the present review, we will discuss the most common pitfalls on PSMA- and SSTR-targeted PET/CT, briefly introduce PSMA- and SSTR-RADS, and define a potential future role of the umbrella framework MI-RADS compared to other classification systems.

## Introduction

Radiotracers with potential theranostic implications are increasingly at the forefront of oncology [1–4]. Prostate-specific membrane antigen (PSMA)-targeted positron emission tomography (PET) imaging agents, labeled with either ^68^Ga or ^18^F, for prostate cancer (PCa) or somatostatin receptor (SSTR)-targeted probes for imaging neuroendocrine tumors (NETs) are already widely utilized and in a variety of clinical contexts [5–9]. While these radiotracers have been relatively rapidly adopted, PSMA- or SSTR-targeted PET/computed tomography (CT) scans must interpreted with caution owing to potential diagnostic pitfalls [10, 11]. However, based on the evaluation of those scans, the nuclear medicine physician or radiologist often has to recommend specific courses of action including whether PSMA-targeted- or peptide receptor radionuclide therapy/radioligand therapy (PRRT/PRLT) would be appropriate for the patient. As such, an exact and precise interpretation of PSMA- and SSTR-PET/CTs is of utmost importance to maximize the full potential of the theranostic promise.

In this regard, several standardized framework systems for the interpretation of radiotracers with potential theranostic implications have been introduced, such as the PROstate cancer Molecular Imaging Standardization Evaluation (PROMISE), the European Association of Nuclear Medicine and Molecular Imaging and Society of Nuclear Medicine and Molecular Imaging joint procedure guideline for PSMA-targeted PET (EANM), and the NeuroEndocrine Tumor Positron Emission Tomography (NETPET) grading system for NETs [12–14]. However, these systems are highly specific and meant to be exclusively applied to one class of imaging agents, potentially requiring the interpreting imaging specialist to be familiar with multiple non-overlapping standardization frameworks. In analogy to the cross-system similarities inherent in different reporting and data systems (RADS) for specific organs such as TI-RADS for thyroid, BI-RADS for breast, or PI-RADS for prostate [15–17], our group has focused on creating RADS for molecular oncology imaging applications, namely PSMA-RADS and SSTR-RADS, that are based on the same fundamental framework [18–20]. Consolidated under an umbrella term, both systems are herein referred to as molecular imaging RADS (MI-RADS). Of note, MI-RADS systems can be applied reciprocally, i.e., if a reader is familiar with one system, the other system can be readily understood, as well. PSMA- and SSTR-RADS should increase the reader’s level of confidence, facilitate communication with other specialists like urologists or gastroenterologists, and may guide the reader as to whether PRRT/PRLT should be considered. In addition, they should assist the observer in interpretation of equivocal findings and guide the clinician in performing appropriate workup of incidental findings. In the present review, we will provide a brief overview of common pitfalls while reading PSMA- and SSTR-PET scans, introduce both RADS systems for molecular oncology imaging, demonstrate how those systems may navigate the reader through indeterminate findings, and, finally, discuss potential future applications.

## Pitfalls on PSMA- and SSTR-targeted PET/CT

### Pitfalls on PSMA-targeted PET/CT

**Fig. 1:**
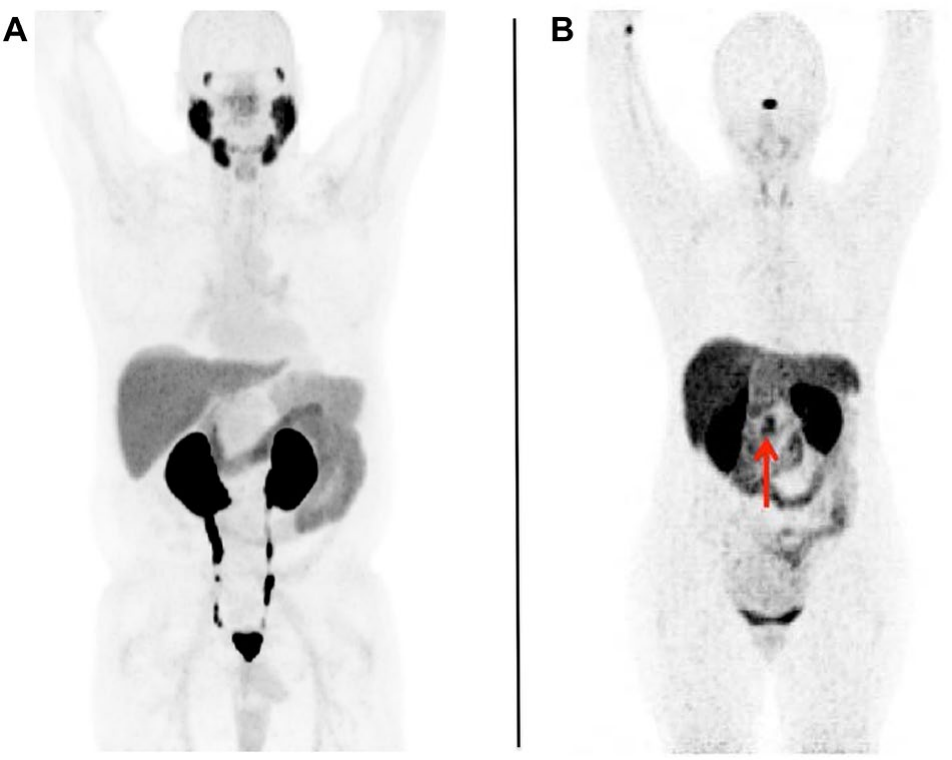
Whole-body maximum intensity projection images with normal biodistribution of **a** prostate-specific membrane antigen (PSMA)-targeted positron emission tomography (PET) (using [18F]-DCFPyL) and **b** somatostatin receptor (SSTR)-targeted PET (using [68Ga]-labeled 1,4,7,10-tetraazacyclododecane-*N,N*′,*N*′′,*N*′′′-tetraacetic acid-d-Phe(1)-Tyr(3)-octreotide ([68Ga]DOTATOC)). No abnormal radiotracer uptake can be appreciated. In both RADS systems (PSMA- and SSTR-RADS), these would be classified as RADS-1A [18–20]. For **a** PSMA-targeted PET, the normal biodistribution includes radiotracer uptake in the lacrimal glands, salivary glands, liver, spleen, kidneys, and small bowel. In addition, radiotracer is seen being excreted within the urinary tract. Modified from Rowe et al. [19], © by the Society of Nuclear Medicine and Molecular Imaging, Inc. For **b** SSTR-targeted PET, the normal biodistribution includes radiotracer uptake in the pituitary gland, major salivary glands, thyroid, adrenal glands, liver, and spleen. Similar to PSMA-targeted PET, radiotracer excretion via the urinary tract can be appreciated. The arrow indicates physiological normal variant uptake in the uncinate. Modified from Werner et al. [20], © by the Society of Nuclear Medicine and Molecular Imaging, Inc.

The normal biodistribution of PSMA radiotracers includes the lacrimal glands, salivary glands, liver, spleen, kidneys, and small bowel. Moreovoer, many PSMA-targeted imaging agents are excreted via the urinary tract and can be visible in the ureters and urinary bladder (Fig. 1a) [19]. Furthermore, PSMA expression is not limited to PCa, but is also expressed in a variety of benign and malignant conditions [10]. Of note, in nervous tissue, PSMA is referred to as glutamate carboxypeptidase II or N-acetylated-alpha-linked acidic dipeptidase (NAALADase), and thus, PSMA has been considered a potential therapeutic target in neurology [21]. Not surprisingly, PSMA-targeted radiotracers have been described to accumulate in ganglia, with the most frequent sites in lumbar ganglia, followed by cervical, stellate, celiac, and sacral ganglia [22, 23].

Benign pathologies mimicking PCa include, but are not limited to granulomatous diseases, such as the systemic inflammatory disorder sarcoidosis, Wegner’s granulomatous, and even tuberculosis [24–27]. Benign bone diseases (fibrous dysplasia, healing fractures, and Paget’s disease) [28–30] are often visualized on PSMA-targeted PET scans. Benign tumors of neurogenic origin, such as schwannomas, peripheral nerve sheath tumors, and meningiomas, can also demonstrate discernible radiotracer uptake [31–33]. Nonprostatic PSMA-targeted ligand uptake has also been documented in hemangiomas and benign soft-tissue pathologies, such as desmoid tumors, intramuscular myxomas, and pseudo-angiomatous stromal hyperplasia [34–37].

PSMA-avid tumor other than PCa includes multiple myeloma, papillary or follicular thyroid carcinoma, pancreatic NET, or renal cell carcinoma [38–43]. For further details regarding pitfalls with PSMA-targeted PET probes, please refer to [10].

### Pitfalls on SSTR-PET/CT

For SSTR-targeted PET imaging agents, the following normal organs are known to show radiotracer uptake: the pituitary gland, major salivary glands, thyroid, adrenal glands, liver, spleen, and bowel. Similar to PSMA-targeted imaging agents, SSTR-based imaging probes are also excreted via the urinary tract (Fig. 1b) [20]. Of note, the pancreatic uncinate process can also be a part of the normal biodistribution, most likely as a result of pancreatic polypeptide-containing cells expressing SSTR on their cell surfaces [44]. Knowledge of this potential pitfall is of utmost importance, as such a finding may lead to unnecessary invasive procedures [11]. In addition, splenosis may also demonstrate discernible radiotracer uptake splenules, even those located inside the pancreas, could be misinterpreted as NET uptake sites [45, 46].

In analogy to PSMA-targeted PET, SSTR-targeted PET also shows radiotracer accumulation in inflammatory diseases, such as inflammation in large arteries, sarcoidosis, or in atherosclerotic plaques [47–49]. Moreover, SSTR-targeted radiotracer-avid structures in bone, which are degenerative in nature, may also lead to false-positive findings on SSTR-targeted PET/CT [20]. An instructive case report noted [^68^Ga]-labeled 1,4,7,10-tetraazacyclododecane-*N,N*′,*N*′′,*N*′′′-tetraacetic acid-d-Phe(1)-Tyr(3)-octreotate ([^68^Ga]DOTATATE) uptake in a vertebral hemangioma [50]. Although NETs are usually located in the gastroenteropancreatic tract in the majority of the cases, there are also rare tumor entities, which are subsumed under NET; for example, medullary thyroid carcinoma or other malignancies that primarily arise from neural crest stem cells, such as pheochromocytomas or paragangliomas. Those tumor entities are also typically positive on SSTR-targeted PET scans [51–53]. Of note, there are a variety of non-NET tumors that also show discernible radiotracer uptake, and those include, but are not limited to: meningioma, primary central nervous system lymphoma, breast cancer, and papillary thyroid cancer [11, 51, 54–58].

Table 1 summarizes the normal biodistribution and important pitfalls on PSMA- and SSTR-targeted PET/CT.

**Table 1:** Normal biodistribution of prostate-membrane-specific antigen (PSMA) and somatostatin receptor (SSTR)-targeting positron emission tomography imaging agents as well as important pitfalls, that may be seen with both imaging probes

Imaging agents	PSMA	SSTR
Normal biodistribution	Lacrimal glands Salivary glands Liver Spleen Kidneys Small bowel Ganglia Radiotracer excretion via urinary tract [18, 19]	Pituitary gland Major salivary glands Thyroid Adrenal glands Liver Spleen Pancreatic uncinate process Splenosis, splenunculi Radiotracer excretion via urinary tract [20]
Important pitfalls	Benign pathologies mimicking PCa Granulomatous diseases: sarcoidosis, Wegner’s granulomatous, tuberculosis [24–27] Benign bone diseases Fibrous dysplasia, healing fractures, Paget’s disease [28–30] Benign tumors of neurogenic origins Schwannomas, peripheral nerve sheath tumors, or meningiomas [31–33] Hemangiomas [34] and benign soft-tissue pathologies Desmoid tumors, intramuscular myxoma, and pseudo-angiomatous stromal hyperplasia [35–37] PSMA-avid tumor entities other than PCa Follicular thyroid carcinoma, pancreatic NET, renal cell carcinoma, radio-iodine refractory thyroid carcinoma [38–40, 42]	Inflammatory diseases Large arteries, sarcoidosis, arthero-sclerotic plaques [47–49] Degenerative bone structures [20] Vertebral hemangioma [50] Rare NET tumors Medullary thyroid carcinoma [51] Paraganglioma and pheochromocytoma [52] Non-NET tumors Meningioma, primary central nervous system lymphoma, breast cancer, papillary thyroid cancer [54–57]

The normal biodistribution of both imaging agents can also be appreciated in Fig. 1

*PCa* prostate cancer, *NET* neuroendocrine tumors

### Introduction of MI-RADS: two reciprocal framework systems for PSMA- and SSTR-PET/CT interpretation

#### MI-RADS

Given the multiple known pitfalls of each class of receptor-targeted radiotracers, a framework system that increases the reader’s confidence in separating pathologic from physiological findings and identifying non-PCa or NET sites of radiotracer uptake that may require further evaluation would be of significant value. This applies, once again, in particular to PSMA- and SSTR-targeted imaging probes, as those agents can potentially be applied in a theranostic setting. A false-positive or -negative interpretation may have an immediate impact on succeeding endoradiotherapies with either ^177^Lu or ^90^Y [3, 59]. Thus, summarized under the umbrella term MI-RADS, the two novel framework systems PSMA-RADS and SSTR-RADS will be briefly introduced [18–20]. Both MI-RADS subsystems are based on a five-point scale (from 1 = no evidence of disease and definitively benign to 5 = high certainty that either PCa or NET are present), and both systems refer to the site of disease and the intensity of radiotracer uptake.

#### PSMA-RADS

Spearheaded by Rowe and coworkers, the PSMA-RADS system was introduced in 2018 [18, 19]. PSMA-RADS-1 refers to benign lesions and is separated into the subcategories of PSMA-RADS-1A lesions, which include benign lesions characterized by either biopsy or anatomic imaging without any abnormal uptake (Fig. 1a), and PSMA-RADS-1B which refers to similar sites with abnormal uptake. A common example would be a radiotracer-avid liver lesion, with magnetic resonance imaging (MRI) findings compatible with hemangioma. PSMA-RADS-2 lesions are likely benign and include those lesions, which have low-level uptake (i.e., ≤ bloodpool level) and which are atypical for PCa, e.g., uptake in a bone lesion strongly suggestive to be of degenerative nature. PSMA-RADS-2 differs from PSMA-RADS-1 in the certainty with which a malignant diagnosis can be excluded, with PSMA-RADS-2 representing those lesions where the possibility of a PCa lesion exists, although it is remote (e.g., overlying uptake from an otherwise benign process could mask malignant uptake).

PSMA-RADS-3 is the most complex category and is separated into four different subcategories. PSMA-RADS-3 includes indeterminate lesions, and thus, further workup (e.g., biopsy or follow-up imaging) is often needed. PSMA-RADS-3A describes equivocal uptake (approximately the level of bloodpool) in a soft-tissue site typical for PCa, e.g., a pelvic lymph node. PSMA-RADS-3B lesions include equivocal uptake in bone lesions not specifically atypical for PCa on anatomic imaging (for example, an osteophyte would be atypical for PCa and any uptake in such a structure should be categorized as PSMA-RADS-2, whereas a bone lesion without anatomic correlate would not be atypical for PCa and might be categorized as PSMA-RADS-3B). Follow-up imaging may confirm disease. While the first two PSMA-RADS-3 classifications have rather low-level uptake, PSMA-RADS-3C sites of uptake are often intense, but in an atypical location for PCa, e.g., an avid lung nodule that is discordant to a patient’s low level of serum prostate-specific antigen. PSMA-RADS-3D lesions do not have radiotracer uptake, but anatomic imaging raises suspicion of malignancy, e.g., a neuroendocrine PCa with obvious sites of metastatic disease on CT but no associated PSMA-targeted radiotracer uptake.

**Fig. 2:**
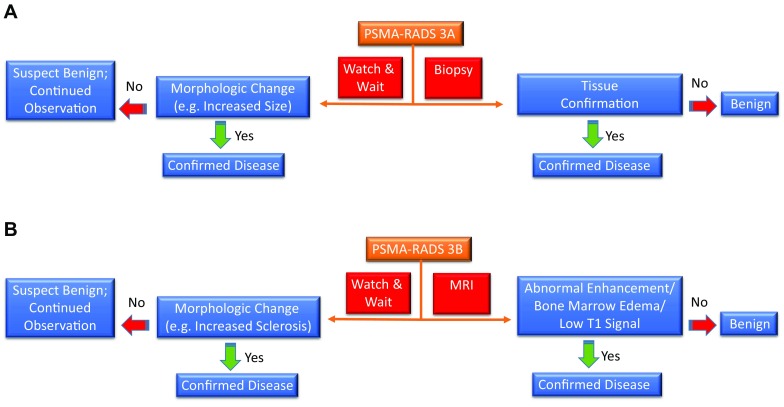
Flowchart for prostate-specific membrane antigen reporting and data system (PSMA-RADS)-3A (**a**) and -3B (**b**) lesions. Appropriate next steps are indicated for such indeterminate lesions [soft-tissue site, PSMA-RADS-3A (**a**) and bone, PSMA-RADS-3B (**b**)]. *MRI* magnetic resonance imaging

**Fig. 3:**
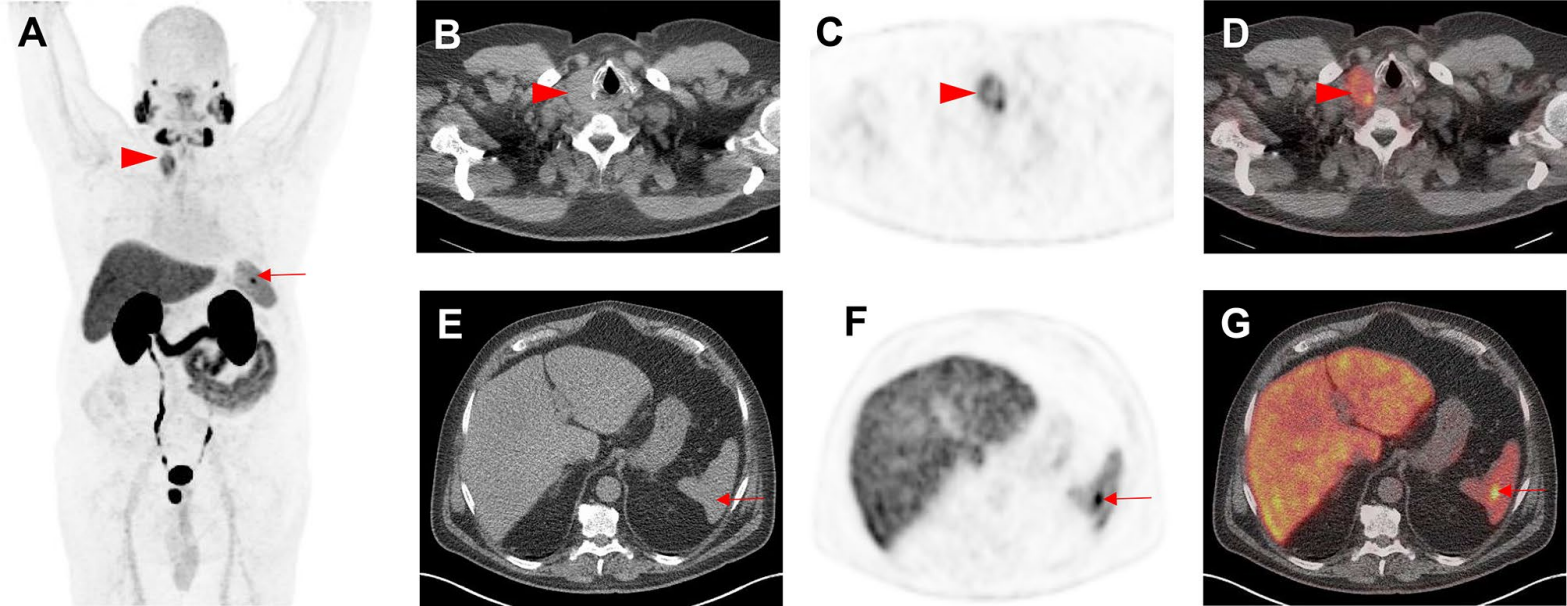
Indeterminate findings on prostate-specific membrane antigen (PSMA)-targeted positron emission tomography (PET)/computed tomography (CT). A 62-year-old man with history of biochemical recurrence undergoing [^18^F]-DCFPyL PET/CT. **a** Whole-body maximum intensity projection image demonstrates multiple foci of radiotracer uptake (red arrowhead and arrow). **b** Axial CT, **c** axial PET, and **d** axial PET/CT demonstrate intense radiotracer in a thyroid nodule (red arrowheads), which was classified as PSMA-RADS-3C and further workup (biopsy) was recommended. Subsequent biopsy yielded papillary thyroid carcinoma. **e** Axial CT, **f** axial PET, and **g** axial PET/CT show focal, intense radiotracer uptake in the spleen (red arrows). This was classified by an experienced reader as a likely inflammatory finding that was not suspicious for either prostate cancer or another malignancy and was labeled as PSMA-RADS-2. The overall scan score for this patient was, therefore, PSMA-RADS-3C

**Fig. 4:**
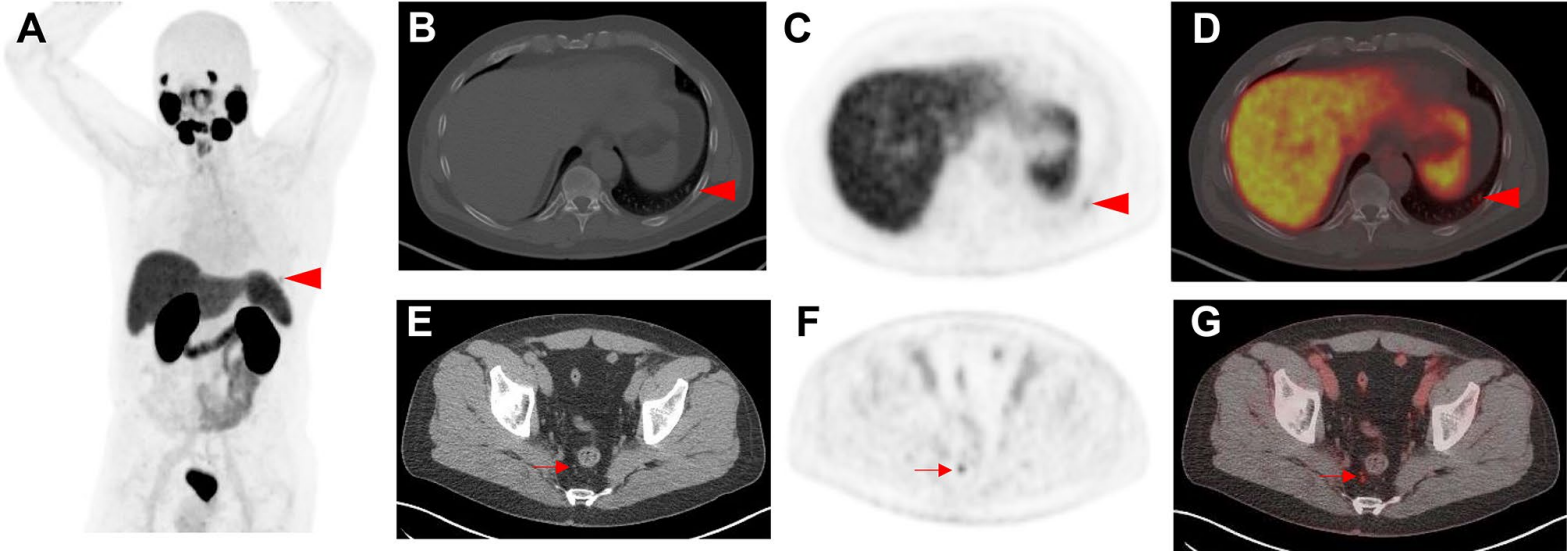
Indeterminate findings on prostate-specific membrane antigen (PSMA) positron emission tomography (PET)/computed tomography (CT). A 58-year-old man with history of biochemical recurrence undergoing [^18^F]-DCFPyL PET/CT. **a** Whole-body maximum intensity projection image demonstrates mild radiotracer uptake in a left-sided rib (arrowhead). **b** Axial CT, **c** axial PET, and **d** axial PET/CT also demonstrate the mild radiotracer uptake at this site (red arrowheads), which was classified as indeterminate for prostate cancer (PSMA-RADS-3B). **e** Axial CT, **f** axial PET, and **g** axial PET/CT showing moderate radiotracer uptake in a right peri-rectal lymph node (red arrows). As this site of radiotracer uptake does not show a corresponding pathologically enlarged lymph node on **e** axial CT, this finding was classified as PSMA-RADS-4. The overall scan score was, therefore, also PSMA-RADS-4

PSMA-RADS-4 describes those lesions with intense uptake in sites highly typical for PCa, but lacking definitive evidence of disease on anatomic imaging. PCa is highly likely with PSMA-RADS-4. PSMA-RADS-4 and -5 differ in their findings on conventional imaging: the latter classification also has intense radiotracer uptake, but corresponding findings can be appreciated on anatomic imaging modalities as well. PCa is almost certainly present [19]. In particular, PSMA-RADS-3A and -3B lesions are of indeterminate nature but suspicious for sites of PCa and, therefore, a workflow chart for those lesions is provided in Fig. 2. Figures 3 and 4 apply PSMA-RADS to PSMA-targeted PET/CTs acquired using [^18^F]-DCFPyL.

#### SSTR-RADS

Intentionally, SSTR-RADS was based on a similar structure to its predecessor, PSMA-RADS, but it takes NET and SSTR-specific details into account. In contrast to the RADS system for PCa molecular imaging, SSTR-RADS also introduced a three-point qualitative assessment scoring, which refers to uptake in normal organs as internal references: uptake level 1 (focal uptake, but ≤ blood pool) through uptake level 2 (> blood pool, but ≤ physiologic liver uptake) to uptake level 3 (> physiologic liver uptake).

In brief, SSTR-RADS-1A refers to normal biodistribution (Fig. 1b), while SSTR-RADS-1B includes those lesions which are benign (characterized by biopsy or anatomical imaging) but with increased abnormal radiotracer uptake (level 2–3). A common example would be prostatitis or benign prostatic hyperplasia. SSTR-RADS 2 lesions are likely benign and are described as soft-tissue sites or bone lesions atypical for NET with an uptake level of 1, e.g., SSTR-avid axillary lymph nodes or uptake in bone lesions suggestive to be of degenerative nature.

Analogous to PSMA-RADS-3 lesions, SSTR-RADS-3 lesions require increased attention and further workup (e.g., biopsy and imaging follow-up). SSTR-RADS-3A and -3B lesions are suggestive of, but not definitive for, NET. For SSTR-RADS-3A, equivocal uptake (uptake level, 1–2) is seen in soft-tissue sites typical for NET metastases, such as low-level uptake of 1 in a mesenteric lymph node. This also applies to SSTR-RADS-3B lesions, but those include uptake in bone lesions not atypical for NET, e.g., uptake level 2 in a rib lesion that is not clearly a fracture. As mentioned earlier, follow-up imaging after 3–6 months (with a shorter interval in terms of increased Ki67) is needed and a biopsy may make findings definitive. In contradistinction to SSTR-RADS-3A or -3B, SSTR-RADS-3C lesions have an uptake level of 3, but in a site highly atypical for NET, i.e., those lesions are suggestive of an SSTR-expressing, non-NET benign tumor or malignant process. SSTR-RADS-3D lesions are not SSTR-avid and can only be appreciated on conventional imaging. SSTR-RADS-3D classified lesions have a high likelihood for either a non-NET malignancy or dedifferentiation of NET lesions. The most common example would be single, dedifferentiated liver lesions, which are SSTR-negative, but highly 2-deoxy-2-[^18^F]-fluoro-d-glucose ([^18^F]-FDG) positive [60]. Tissue confirmation should be considered, so that potential tumor escape can be ruled out.

**Fig. 5:**
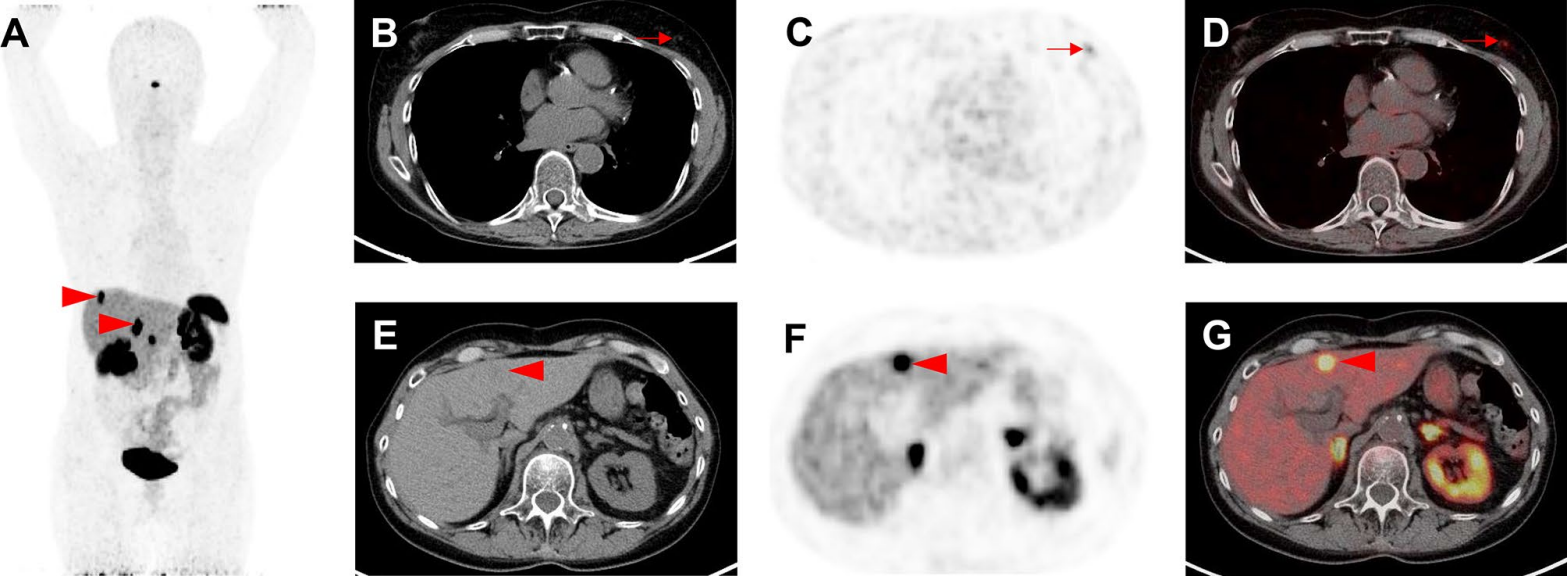
Indeterminate findings on somatostatin receptor (SSTR) positron emission tomography (PET)/computed tomography (CT). **A** 63-year-old man with history of a gastroenteropancreatic neuroendocrine tumor, who underwent [^68^Ga]-labeled 1,4,7,10-tetraazacyclododecane-*N,N*′,*N*′′,*N*′′′-tetraacetic acid-d-Phe(1)-Tyr(3)-octreotide ([^68^Ga]-DOTATOC) PET/CT for staging. **a** Whole-body maximum intensity projection image demonstrates suspicious radiotracer uptake (red arrowheads) in the liver. **b** Axial CT, **c** axial PET and **d** axial PET/CT show mild radiotracer uptake in the left breast (red arrows). This has been classified as SSTR-RADS-3C by an experienced reader. **e** Axial CT, **f** axial PET, and **g** axial PET/CT demonstrate intense radiotracer uptake in multiple liver lesions (one of which is demarcated by the red arrows). As there is subtle hypoattenuation in the liver on **e** axial CT, this finding was classified as SSTR-RADS-5 and, therefore, the overall SSTR-RADS score was 5. Based on this scoring, peptide receptor radionuclide therapy should be considered [20]

SSTR-RADS-4 are those lesions which are highly SSTR-avid in a site typical for NET, but lacking evidence corresponding malignant finding on the conventional imaging. SSTR-RADS-5 also includes highly SSTR-avid lesions in a site typical for NET, but the conventional imaging demonstrates a corresponding finding. Both categories have an uptake level of 3, and while, for SSTR-RADS-4, NET is highly likely, it is almost certainly present for SSTR-RADS-5. Of note, PRRT is recommended for both SSTR-RADS-4 and -5 classifications, but common recommendations of practical guidelines still apply [61]. For SSTR-RADS-3B, PRRT may be an option for increased number of lesions, but single lesions should be treated by a locoregional procedure, e.g., selective internal radiotherapy [20, 62, 63]. For SSTR-RADS-3D lesions, a combined treatment approach may be also applicable, e.g. treating a dedifferentiated lesion with a locoregional procedure, but an increased number of remaining SSTR-avid lesions may benefit from PRRT as well [20]. Figure 5 applies SSTR-RADS to an SSTR-PET/CT using [^68^Ga]-labeled 1,4,7,10-tetraazacyclododecane-*N,N*′,*N*′′,*N*′′′-tetraacetic acid-d-Phe(1)-Tyr(3)-octreotide ([^68^Ga]DOTATOC).

Of note, both MI-RADS (PSMA- and SSTR-RADS) systems can define an overall RADS score. In addition, a reader may choose up to five target lesions (most intense in uptake and largest in size) to individually demarcate. The highest scored lesion takes priority over the other lesions to indicate an overall scan impression (e.g., if one target lesion has been classified as SSTR-RADS-3A and a second lesion is rated as SSTR-RADS 5, the overall scan score would be SSTR-RADS-5). If the overall scan impression is designated as SSTR-RADS-4 or -5, endoradiotherapy may be highly recommended [20].

Table 2 provides a head-to-head comparison of both RADS systems in molecular oncology imaging, along with recommendations for workup or consideration for PRRT/PRLT. Table 3 indicates the minimum required information (patient’s history and imaging data) in a PSMA- or SSTR-targeted PET/CT report.

**Table 2:** Head-to-head comparison of both reporting and data systems (RADS) in molecular imaging (MI-RADS), which are prostate-membrane-specific antigen (PSMA)-RADS and somatostatin receptor (SSTR)-RADS

MI-RADS classification		PSMA- and SSTR-RADS [18, 20]	Workup	Uptake level^a^	PRRT/PRLT?
1	1A	Benign lesion, characterized by biopsy or anatomic imaging without abnormal uptake	n/a	1	N
	1B	Benign lesion, characterized by biopsy or anatomic imaging with abnormal uptake	n/a	2–3	N
2		Soft-tissue site or bone lesion atypical for metastatic PCa or NET	n/a		N
3	3A	Equivocal uptake in soft-tissue lesion typical of PCa or NET	B, F/U	1–2	N
	3B	Equivocal uptake in bone lesion not atypical of PCa or NET	B, F/U	1–2	N^b^
	3C	Intense uptake in site highly atypical of all but advanced stages of PCa or NET (i.e., high likelihood of nonprostatic/non-NET malignancy or other benign tumor)	B	3	N
	3D	Lesion suggestive of malignancy on anatomic imaging but lacking uptake. For SSTR-RADS: 2-deoxy-2-[^18^F]-fluoro-D-glucose [^18^F]-FDG is recommended to rule out potential dedifferentiation of a single lesion	B, F/U	Not available	N^b^
4		Intense uptake in site typical of PCa or NET but lacking definitive findings on conventional imaging	n/a	3	Y
5		Intense uptake in site typical of PCa or NET but with definitive findings on conventional imaging	n/a	3	Y

*PRRT* peptide receptor radionuclide therapy, *PRLT* PSMA-targeted radioligand therapy, *PCa* prostate carcinoma, *n/a* not applicable, *N* no (endoradiotherapy not recommended), *NET* neuroendocrine tumors, *B* biopsy, *F/U* follow-up imaging (3–6 months, e.g., depending on Ki67 in NET), *Y* yes (endoradiotherapy recommended)

^a^Applies only to SSTR-RADS

^b^In terms of a single lesion, a locoregional procedure may be preferred, while, for increasing lesions, PRRT may be applicable

**Table 3:** Minimum required data in a clinical report for interpreting either a prostate-specific membrane antigen (PSMA) positron emission tomography (PET)/computed tomography (CT) or somatostatin receptor (SSTR) PET/CT [20]

	PSMA-PET/CT	SSTR-PET/CT
Patient’s history	Date of tumor biopsy Date of primary diagnosis Previous therapies Previous conventional or functional imaging History and treatment of other malignancies	
	Laboratory test results (PSA) Gleason score	Primary tumor origin Laboratory test results (CgA, NSE) Ki67/proliferation index Previous [^18^F]-FDG scans
Imaging data	Injected amount/activity Uptake time Field of view	

*PSA* prostate-specific antigen, [^*18*^*F*]-*FDG* 2-deoxy-2-[^18^F]-fluoro-d-glucose, *CgA* chromogranin A, *NSE* neuron-specific enolase

### MI-RADS: a flexible system that can accommodate new information and novel radiotracers

The MI-RADS frameworks require validated in prospective studies. For example, it is critical to establish whether different readers with different levels of experience in either reading PSMA- or SSTR-PET/CT scans arrive at the same conclusions, while, applying RADS for molecular imaging, interobserver agreement studies should be performed. Ideally, multiple study sites should be included. Fendler et al. have conducted those studies in PSMA- and SSTR-targeted PET using ^68^Ga-labeled imaging agents, but a specific framework system has not been used. Nonetheless, a high interobserver agreement rate has been demonstrated and thus, one may expect comparable findings while applying structured reporting systems, such as MI-RADS [64, 65].

Another area of validation is the need to follow MI-RADS-3A and -3B lesions to determine the rates at which those indeterminate findings are ultimately found to harbor PCa or NET. Accordingly, this would increase the reader’s level of confidence about appropriate recommendations to make. In addition, MI-RADS should be applied to outcome studies, and the herein proposed standardization for both PSMA- and SSTR-targeted PET may open avenues for multicenter trials, e.g., to investigate which lesions are associated with poorer outcome (e.g., a high number of MI-RADS-5 lesions may be linked to worse overall or progression-free survival). Moreover, the workup and treatment recommendations based on MI-RADS should be critically reviewed and further validated. We note that MI-RADS is a homonym for “My-RADS”, suggesting the potential role of a system such as this for personalized medicine and guiding individualized therapies.

Notably, SSTR-RADS is the first proposal for PRRT based on PET, as the commonly used Krenning Score had been initially invented for single-photon emission computed tomography using SSTR imaging agents (Octreoscan) [66]. While the Krenning Score takes planar images into account to define whether a patient is suitable for PRRT, SSTR-RADS-based treatment recommendations refer to the far more complex evaluation of an entire SSTR-PET/CT scan. However, the Krenning Score is well established and SSTR-RADS has to still prove its robustness in patient selection for PRRT [67]. Apart from that, the role of MI-RADS has to be constantly refined and improved upon. There are other systems that address the need of standardization in receptor-based PET imaging, such as PROMISE, the EANM Consensus Paper, or the NETPET Grade [12–14]. While all of those systems include a huge variety of different parameters, MI-RADS is easy to memorize and readily applicable [19, 20]. Thus, MI-RADS may serve as a robust tool in clinical practice, while framework systems like PROMISE can provide details that are necessary to enrich data sets in a research setting [12]. Nonetheless, the concept of MI-RADS includes two systems, which are reciprocable, and thus, if one system has been understood, the reader may use the other framework system with only small adjustments as well. Apart from that, the concept of MI-RADS is a living framework system for molecular oncology imaging. Therefore, it could potentially be expanded to other theranostic PET radiotracers, such as the theranostic twins for C–X–C chemokine receptor CXCR4-directed [^68^Ga]-Pentixafor/[^177^Lu]-Pentixather (“CXCR4-RADS”), the Fibroblast Activation Protein (FAP) inhibiting [^68^Ga]-/[^90^Y]-FAPI04 (“FAPI-RADS”), or ^177^Lu-labeled, gastrin releasing peptide receptor (GRPR) targeting bombesin peptides (“GRPR-RADS”) [4, 68–70]. MI-RADS cannot replace the imaging specialist needing to possess disease-specific knowledge to appropriately categorize findings; however, the modular nature and significant overlap of the subsystems within MI-RADS still minimize the effort necessary to apply this over-arching framework.

## Conclusions

The use of PSMA and SSTR-PET imaging agents is currently expanding outside of the confines of clinical studies [2, 5]. In the course of using these agents, a manifold of pitfalls has been discovered. Adding to the complexity of those potential false-positive or -negative discoveries, some of those pitfalls may apply to both imaging probes (e.g., sarcoidosis), but certain false findings may exist only for one of those PET agents (e.g., uptake in nervous tissue for PSMA-PET) [10, 11, 22, 23, 71]. In this regard, a great deal in progress has been made by the introduction of several framework systems for theranostic PET radiotracers [12–14]. Notably, the proposed concept for standardization in molecular imaging, entitled MI-RADS, represents a harmonization of the interpretation and reporting of the two most commonly used theranostic PET imaging probes, namely PSMA- and SSTR-RADS. As a major advantage to MI-RADS over other previously described classification systems is that PSMA-RADS and SSTR-RADS, these are the only systems to date that can be applied reciprocally [18–20]. Of note, those systems take disease- and radiotracer-specific details into account and indeterminate findings are critically reviewed, along with treatment and workup recommendations. Thus, MI-RADS may help to increase the reader’s level of confidence in interpreting PSMA- or SSTR-PET/CTs, it may serve as a platform to communicate with referring clinicians and it gives practical advice of appropriate next steps, which may be critical in a busy clinical PCa and/or NET practice. Nonetheless, future studies should focus on the validation of these systems.
